# Supplementary material for: MetaRibo-Seq measures translation in microbiomes
Source: Nat Commun. 2020 Jun 29;11:3268. doi: 10.1038/s41467-020-17081-z (PMC7324362; doi:10.1038/s41467-020-17081-z)
Supplement: Supplementary file 10 — Supplementary Data 7 [file 41467_2020_17081_MOESM10_ESM.zip › File2/Confidence_High_Taxonomy/36979_out.krona.html]

Javascript must be enabled to view this page.

members
magnitude
magnitudeUnassigned
count
unassigned
taxon
rank

36979\_out

2

superkingdom
2
2

1239
2
phylum

91061
2
class

186826
2
order

2
1300
family

genus
2
1301

1

SRS104087\_contig\_number\_contig-100\_351.352
113107
species

species
1

SRS017814\_contig\_number\_3251
1305
